# Diagnostic accuracy of history taking, physical examination, and auxiliary examination for thumb osteoarthritis: a systematic review

**DOI:** 10.1080/07853890.2025.2524086

**Published:** 2025-06-26

**Authors:** Yisha He, Patrick Krastman, Sita M. A. Bierma-Zeinstra, Gerald Kraan, Nina M. Mathijssen, Jos Runhaar

**Affiliations:** a Department of General Practice, Erasmus MC University Medical Center Rotterdam, Rotterdam, Netherlands; b Department of Orthopedic and Sports Medicine, Erasmus MC University Medical Center Rotterdam, Rotterdam, Netherlands; c Department of Orthopaedic Surgery, Reinier de Graaf Groep, Delft, Netherlands

**Keywords:** Thumb, osteoarthritis, diagnosis, systematic review

## Abstract

**Objective:**

To conduct a systematic review to evaluate the diagnostic accuracy of history taking, physical examination, and auxiliary examination for thumb osteoarthritis (OA).

**Methods:**

MEDLINE ALL, EMBASE, Cochrane Central Register of Controlled Trials, Web of Science Core Collection, and CINAHL were searched up to October 2023. Studies focused on patients with hand or thumb pain suspected of thumb OA, considering any diagnostic methods as the index test, with any diagnostic assessment of thumb OA as the reference standard were included. Sensitivity, specificity, positive predictive value, negative predictive value, and diagnostic accuracy were extracted. QUADAS and GRADE were applied to assess the evidence.

**Results:**

Seven studies were included. History taking (two studies, 1096 participants) showed very low certainty of evidence;physical examination (five studies, 427 participants) revealed moderate certainty of evidence; auxiliary examinations (one study, 20 participants) indicated low certainty of evidence. History taking showed sensitivity of 47% to 100%, specificity of 40% to 63%, and accuracy of 40% to 80%. Physical examination demonstrated sensitivity of 2% to 100%, specificity of 75% to 100%, and accuracy of 47% to 98%. Auxiliary examination exhibited sensitivity of 72%, specificity of 86%, and accuracy of 76%. The adduction test, extension test, and metacarpal pressure-shear tests were reported to have accuracy above 90% based on two studies.

**Conclusions:**

Based on few studies, the diagnostic accuracy of history taking and physical examination for thumb OA varied across studies, while knowledge about auxiliary examination was limited. The adduction test, extension test, and metacarpal pressure-shear test are recommended for thumb OA diagnosis.

## Introduction

Similar to osteoarthritis (OA) in other joints, thumb OA can cause pain, decreased joint stability, and diminished strength [1]. Given that the thumb plays a significant role in hand and upper limb function [2], thumb OA greatly affects patients’ quality of life [3].

The reported prevalence of thumb OA varies widely [4,5], with indications that it increases with age [5]. With the global trend of an aging population [6], the estimated health burden associated with thumb OA is expected to gradually rise [7].

Accurate and timely diagnosis is crucial for the management of thumb OA and aims to alleviate pain, maintain optimal function, and slow disease progression. In clinical practice, history taking, physical examination, and auxiliary examinations are utilized for the diagnosis of thumb OA. There is currently no universally recognized diagnostic gold standard for identifying thumb OA [8,9]. Historically, the clinical diagnosis of thumb OA commonly relied on patients’ reports of thumb pain and joint stiffness, combined with radiographic evidence of structural changes (radiographic osteoarthritis (ROA)). Similar to hand OA [10], in thumb osteoarthritis there is a discrepancy between the structural changes in the joint visible on imaging and the clinical symptoms of thumb OA reported by patients [11,12].

Numerous studies have investigated history taking, physical examination, and auxiliary examinations as diagnostic approaches of thumb OA [8,13–23], some of these have reported data regarding diagnostic accuracy [13,15–17,20,22]. These articles provide valuable evidence regarding these diagnostic measures. However, there is currently no systematic overview of the accuracy of diagnostic approaches for thumb OA. Therefore, this study aimed to conduct a systematic review to evaluate the diagnostic accuracy of history taking, physical examination, and auxiliary examinations for diagnosing thumb OA. Additionally, it aimed to determine the most accurate diagnostic method or combination of methods, assessed the quality and certainty of evidence, and evaluated potential biases and sources of variation within the current literature.

## Methods

This study was conducted according to the Preferred Reporting Items for Systematic Reviews and Meta-Analyses (PRISMA) guidelines [24–26]. The PRISMA-DTA checklist and PRISMA-S checklist can be found in Online Appendices A and B. The flowchart was created according to PRISMA-2020. Prior to the initiation of this study, the research question, search strategy, inclusion and exclusion criteria were established and documented in the protocol, which has been registered on PROSPERO (Registration number: CRD42022309959).

### Search strategy

Based on the research question, a search strategy was created, in consultation with an expert librarian from the Erasmus MC Medical Library. Databases searched included Embase, MEDLINE ALL, Web of Science Core Collection, Cochrane Central Register of Controlled Trials, and CINAHL. Search terms included ‘thumb’, ‘OA’, ‘interphalangeal joint’, ‘metacarpophalangeal joint’, ‘carpometacarpal joint’, ‘scaphotrapeziotrapezoid joint’, ‘history taking’, ‘physical examination’, ‘auxiliary examination’, and their various synonyms, which encompass various search terms such as ‘Kapandji’, ‘goniometry’, ‘imaging’, ‘ultrasound’, ‘MRI’, ‘SE’, ‘SP’, and ‘reliability’. The last search was performed on October 5, 2023. The complete search strategy is provided in the Online Appendix C.

### Study selection

Inclusion criteria:

1. Participants: patients with hand or thumb pain, suspected of having thumb OA.
2. Index test: history taking, physical examination, auxiliary examinations, or any other methods for diagnosing thumb OA.
3. Reference standard: any diagnostic assessment of thumb OA, such as radiological classification, clinical assessment, expert consensus, or a combination of two or more of the above.
4. Sensitivity (Se), specificity (Sp), positive predictive value (PPV), negative predictive value (NPV), or diagnostic accuracy is reported or can be calculated.

Exclusion criteria:

1. Study type: case report, review, letter, comment, conference proceeding, case-control study.
2. Cadaver or animal study.

The screening of titles and abstracts was independently conducted by two reviewers (YH and JR). Articles with disagreements were discussed to reach a consensus. The articles that passed the title and abstract screening proceeded to full-text screening, which was also independently conducted by the same two reviewers. Again, articles with disagreements were discussed to reach a consensus.

### Data extraction

The extracted data included Se, Sp, PPV, NPV, diagnostic accuracy, the publication years, study design, sample size, region of the study populations, and the specific joint of the thumb studied. If diagnostic values, meaning Se, Sp, PPV, NPV, and accuracy, were not reported, relevant data required to calculate these were extracted from the article. Diagnostic accuracy was calculated using the formula: diagnostic accuracy = (the number of true positives + the number of true negatives)/the total number of subjects. Data extraction and calculation were independently performed by YH and JR. The results were cross-checked to ensure consistency. For any discrepancies, YH and JR extracted the data together to reach a consensus.

### Quality assessment

Methodological quality assessment was conducted using the Quality Assessment of Diagnostic Accuracy Studies tool (QUADAS-2) [27]. Quality assessments were performed independently by YH and JR. Any disagreement was resolved by discussion. Certainty of evidence was assessed using the Grading of Recommendations Assessment, Development and Evaluation (GRADE) approach [28].

## Results

### Description of the included studies

**Figure 1.**
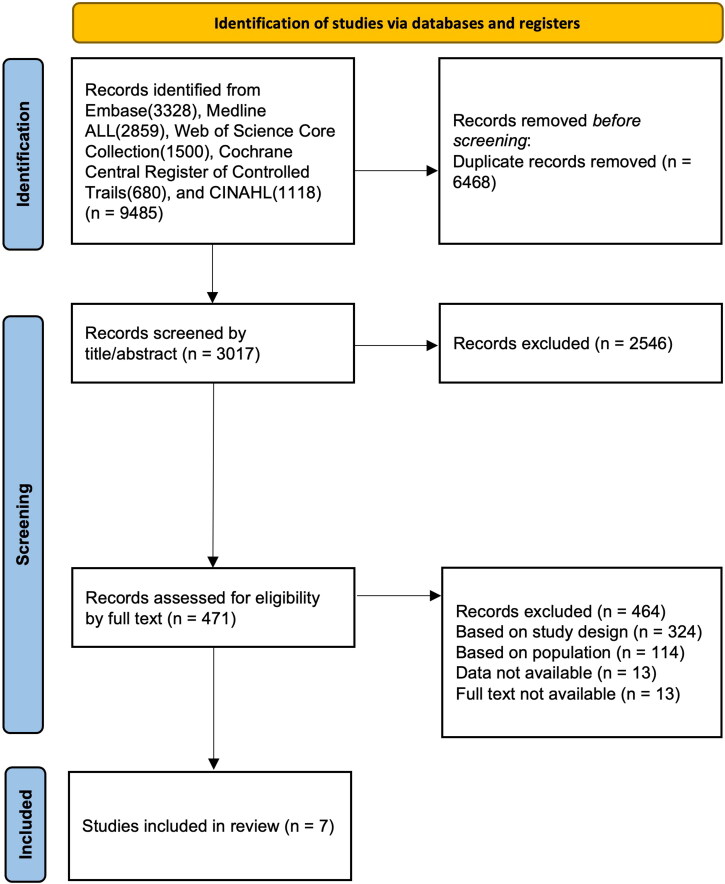
Flowchart of literature search and screening process.

The search strategy retrieved a total of 9485 articles. EMBASE results were 3328, MEDLINE ALL results were 2859, Web of Science Core Collection results were 1500, Cochrane Central Register of Controlled Trials results were 680, and CINAHL results were 1118. After title and abstract screening, 471 articles were selected, of which 7 were deemed eligible after full text screening [13,15–17,20,22,29]. The flowchart of the literature search and screening process is shown in Figure 1. The extracted data is presented in a 2 × 2 table, as shown in Online Appendix F.

The publication years, study designs, setting and region, population size and age, the joint of the thumb studied, assessed index tests, and selected reference standards are listed in Table 1.

**Table 1.** Basic information for eligible studies (*N* = 7).

Author, Year	Study design	Setting and region	Population size and age	Joint studied	Index Test	Reference Standard
Arnold et al. 2022 [5]	Prospective	urban hospital in the Northeastern United States	68, Age mean 66.4(SD 11.8)	CMC I + STT	Grind Test/Seesaw Test	X-ray (Eaton stage 2–4)
Gelberman et al. 2015 [13]	Prospective cross-sectional study	two outpatient offices at a tertiary institution, US	139, Age range (25–68)	CMC I	Adduction/ Extension/Grind Test/ Basal Joint Tenderness/ Eichoff Test/ STT tenderness/ Radioscaphoid Tenderness/ First Dorsal Compartment Tenderness/ MCP Tenderness/ A1 Pully Tenderness	history + physical examination +X-ray (Eaton stage 2–4)
Merritt et al. 2010 [27]	Prospective convenience sample study	three private orthopedic clinics, one occupational medicine clinic, and the general community located in US	54, Age mean 59.5(SD 13.4, range 23–91)	CMC I	Grind Test	X-ray (Eaton stage 1–4)
Sela et al. 2019 [34]	Retrospective	University of Pittsburgh Medical Center, Orthopaedic Surgery, Community Medicine, US	104, Age mean 59, range 22–91	CMC I	Grind Test/ MC Pressure-shear Test/ MC Flexion Test	X-ray (Eaton stage 1–4)
Komatsu et al. 2017 [18]	Retrospective	Department of Orthopaedic Surgery, Shinshu University School of Medicine, and Center of Osteoporosis and Spinal Disorders, Kamimura Orthopaedic Clinic, Matsumoto, Japan	20, Age mean 64(SD 13.29)	CMC I + ST	MRI bone signal change/Female	X-ray (Eaton stage 2–4)
Kwok et al. 2014 [20]	Prospective population based observational cohort	Hand (CAS-HA) and Knee (CAS-K) cohort, UK	1076, Age mean 64.7 (SD 8.3)	CMC I + ST	Manual Occupation/Female/TB Pain	X-ray (K-*L* > 2 in at least one CMC I or STJ)/TB pain + X-ray (K-*L* > 2 in at least one CMC I or ST)/ X-ray (Verbruggen-Veys stage erosive/ remodelled in the CMC I)
Model et al. 2016 [28]	Prospective cohort	Consecutive patients presented to hand surgeons, US	62, Age mean age 62 ± 8, range 45–86	CMC I	Grind Test/ Lever Test/ MP Extension Test/ Palpation	TB pain + X-ray (staging system not described)

CMC I = carpometacarpal first; ST = scaphotrapezium; STT = scaphotrapeziotrapezoid; TB = thumb base; ROA = radiographic osteoarthritis; SOA = symptomatic osteoarthritis; MP = metacarpophalangeal; MC = metacarpal.

Regarding the index test, five out of seven eligible studies specifically assessed the diagnostic accuracy of physical examination [13,15,20,22,29], one study focused solely on the diagnostic accuracy of history taking [17], and one study investigated both history taking and auxiliary examinations [16]. The history taking items studied included female sex [16,17], any thumb base (TB) pain [17], and manual occupation [17]. The physical examination methods investigated included the grind test [13,15,20,22,29], lever test [29], metacarpophalangeal (MP) extension test [29], seesaw test [13], extension test [15,22], adduction test [15], adduction and extension [15], adduction or extension [15], basal joint tenderness [15], palpation [29], Eichoff test [15], scaphotrapeziotrapezoid (STT) tenderness [15], radioscaphoid tenderness [15], first dorsal compartment tenderness [15], MP tenderness [15], A1 pulley tenderness [15], metacarpal (MC) flexion test [22], and MC pressure-shear test [22]. The auxiliary examination studied was the MRI for signal changes [16].

In terms of the reference standard, five studies defined radiographic changes [13,15,16,20,22], while two study incorporated both radiographic changes and TB pain as the reference standard [17,29]. Among these studies, different radiographic staging systems or different stages were used. The selected radiographic standard included Kellgren-Lawrence (KL) grade ≥2 [17], Verbruggen-Veys system E or R stage [17], Eaton and Littler stage or Eaton stage ≥1 [20,22], and Eaton and Littler stage or Eaton stage ≥2 [13,15,16]. One study did not specify which radiological assessment method was used [29].

### Diagnostic accuracy and associated metrics

All seven studies presented Se, Sp, PPV, and NPV. For one study it was not possible to calculate diagnostic accuracy from the reported data, while the remaining six articles presented diagnostic accuracy or it could be calculated from the reported data.

The Se of history taking ranged from 47% to 100%, with a Sp ranging from 40% to 63%. The PPV ranged from 2% to 81%, and the NPV ranged from 42% to 100%. The diagnostic accuracy of history taking ranged from 40% to 80% (See Table 2).

**Table 2.** Diagnostic accuracy and associated metrics.

Index Test	Reference Standard	Sensitivity (Se)	Specificity (Sp)	Positive predictive value (PPV)	Negative predictive value (NPV)	Accuracy	Author, Year
History Taking							
Female sex	X-ray (K-*L* > 2 in at least one CMC I or STJ)	65%	45%	58%	51%	55%	[20]
Female sex	TB pain + X-ray (K-*L* > 2 in at least one CMC I or ST)	69%	43%	35%	76%	51%	[20]
Female sex	X-ray (Verbruggen-Veys stage eroded/ remodeled in the CMC I)	58%	40%	2%	98%	40%	[20]
Female sex	X-ray (Eaton stage 2–4)	94%	43%	81%	75%	80%	[18]
Any TB pain	X-ray (K-*L* > 2 in at least one CMC I or STJ)	62%	51%	60%	53%	57%	[20]
Any TB pain	TB pain + X-ray (K-*L* > 2 in at least one CMC I or ST)	100%	63%	55%	100%	74%	[20]
Any TB pain	X-ray (Verbruggen-Veys stage eroded/ remodeled in the CMC I)	83%	44%	3%	99%	45%	[20]
Manual occupation	X-ray (K-*L* > 2 in at least one CMC I or STJ)	47%	46%	51%	42%	46%	[20]
Manual occupation	TB pain + X-ray (K-*L* > 2 in at least one CMC I or ST)	49%	49%	30%	69%	49%	[20]
Manual occupation	X-ray (Verbruggen-Veys stage eroded/ remodeled in the CMC I)	63%	50%	3%	98%	50%	[20]
Physical Examination							
Grind test	X-ray (Eaton stage 2–4)	13%	91%	43%	66%	63%	[5]
Grind test	X-ray (Eaton stage 2–4)	17%	98%	80%	68%	69%	[5]
Grind test	History + physical examination + X-ray (Eaton stage 2–4)	44%	92%	75%	76%	76%	[13]
Grind test	X-ray (Eaton stage 1–4)	42%	93%	96%	30%	53%	[27]
Grind test	X-ray (Eaton stage 1–4)	53%	80%	91%	32%	59%	[27]
Grind test	X-ray (Eaton stage 1–4)	64%	100%	100%	32%	70%	[34]
Grind test	TB pain + X-ray	41%	100%	100%	26%	–	[28]
Seesaw test	X-ray (Eaton stage 2–4)	71%	82%	68%	84%	78%	[5]
Seesaw test	X-ray (Eaton stage 2–4)	42%	86%	63%	73%	71%	[5]
MC extension test	X-ray (Eaton stage 1–4)	46%	100%	100%	28%	55%	[34]
Extension	History + physical examination + X-ray (Eaton stage 2–4)	94%	95%	90%	97%	94%	[13]
MP extension test	TB pain + X-ray	65%	95%	98%	36%	–	[28]
Adduction	History + physical examination + X-ray (Eaton stage 2–4)	94%	93%	88%	97%	94%	[13]
Adduction and extension	History + physical examination + X-ray (Eaton stage 2–4)	88%	97%	93%	94%	94%	[13]
Adduction or extension	History + physical examination + X-ray (Eaton stage 2–4)	100%	91%	86%	100%	94%	[13]
Basal joint tenderness	History + physical examination + X-ray (Eaton stage 2–4)	94%	81%	73%	96%	86%	[13]
Palpation	TB pain + X-ray	91%	76%	95%	64%	–	[28]
Eichoff test	History + physical examination + X-ray (Eaton stage 2–4)	13%	75%	21%	62%	53%	[13]
STT tenderness	History + physical examination + X-ray (Eaton stage 2–4)	29%	75%	38%	67%	59%	[13]
Radioscaphoid tenderness	History + physical examination + X-ray (Eaton stage 2–4)	17%	77%	28%	64%	56%	[13]
First dorsal compartment tenderness	History + physical examination + X-ray (Eaton stage 2–4)	2%	81%	6%	61%	54%	[13]
MP tenderness	History + physical examination + X-ray (Eaton stage 2–4)	13%	98%	75%	68%	68%	[13]
A1 pulley tenderness	History + physical examination + X-ray (Eaton stage 2–4)	15%	89%	41%	66%	63%	[13
MC pressure-shear test	X-ray (Eaton stage 1–4)	99%	95%	99%	95%	98%	[34]
Lever test	TB pain + X-ray	82%	81%	95%	49%	–	[28]
MC flexion test	X-ray (Eaton stage 1–4)	36%	100%	100%	25%	47%	[34]
Auxiliary Examination							
MRI: Signal change	X-ray (Eaton stage 2–4)	72%	86%	93%	55%	76%	[18]

CMC I = carpometacarpal first; ST = scaphotrapezium; TB = thumb base; MP = metacarpophalangeal; MC = metacarpal.

The Se of physical examination ranged from 2% to 100%, with a Sp ranging from 75% to 100%. The PPV ranged from 6% to 100%, and the NPV ranged from 28% to 100%. The diagnostic accuracy of physical examination ranged from 47% to 98% (See Table 2).

In the single study that assessed auxiliary examination, the Se of was 72%, with a Sp of 86%. The PPV was 93%, and the NPV was 55%. The diagnostic accuracy of MRI was 76% (See Table 2).

### Methodological quality assessment

Regarding patient selection, four studies were rated as having a low risk of bias [15,17,22,29], while the risk of bias was unclear for the remaining three studies [13,16,20]. For the index test domain, five studies had a low risk of bias [13,15,17,20,29], while the risk of bias was unclear for two studies [16,22]. In terms of the reference standard domain, six studies had a low risk of bias [15–17,20,22,29], while one study had a high risk of bias [13]. For the flow and timing domain, four studies had a low risk of bias [15,17,20,29], while the risk of bias was unclear for the other three studies [13,16,22] As for applicability concerns, only one study had an unclear rating in the index test domain [16], while the rest were rated as low [13,15–17,20,22,29]. The results of their QUADAS-2 methodological assessments are presented in Online Appendix D.

### Certainty of evidence

The certainty of evidence is presented in Online Appendix E. For the diagnostic accuracy of history taking for thumb OA, two studies involving a total of 1096 participants were conducted, but the certainty of evidence is very low. For physical examinations, five studies with a total of 427 participants were analyzed, revealing a moderate certainty of evidence. The assessment of auxiliary examinations, based on one study with 20 participants, also indicates low certainty of evidence.

## Discussion

### The current state of evidence

This is the first systematic review analyzing the diagnostic accuracy of history taking, physical examination, and auxiliary tests for the diagnosis of thumb OA among patients with hand or thumb pain, suspected of having thumb OA. Although thumb OA affects patients’ quality of life and that accurate and timely diagnosis is desirable, research into the diagnostic accuracy of diagnostic tests is very limited, with only seven eligible studies identified [13,15–17,22,29]. Some scholars claimed that the CMC is the most commonly affected joint in thumb OA [4], while others argue that it is the IP joint [30]. All studies included in this review assessed the diagnosis of CMC I and ST/STT OA and none IP or MCP OA. Among the seven included studies, only one study investigated MRI [16]. Despite the corresponding advancements in auxiliary examination techniques such as MRI, CT, and US, we did not find any studies meeting the criteria for CT or US in this review. The lack of sufficient evidence hinders our ability to compare the diagnostic accuracy of different auxiliary examinations. Among the seven eligible studies, five utilized structural changes on imaging [12,13,17,20,22], while two study combined structural changes on imaging combined with TB pain as the reference standard [15,29]. Within the studies that solely relied on imaging changes for diagnosis, different classification systems (KL classification system and Eaton stages system) were employed to determine the presence of thumb OA. This corresponds to the current absence of a universally accepted gold standard for diagnosing thumb OA. While many current studies on thumb OA utilize imaging-based criteria, it may not necessarily be the best methodology. Given that symptomatic OA are more strongly associated with increased mortality compared to asymptomatic radiographic OA [31], symptoms should be considered a primary factor in the classification criteria. As shown in Table 1, the settings and designs of the 7 studies are also not identical. This likely results in variations among the study participants. These differences can have a significant impact on the diagnostic performance of the index test.

The methodological quality assessment of the included studies was deemed acceptable. The current body of evidence for these diagnostic tests in Thumb OA is characterized by limitations, indirectness, and inconsistency, resulting in a very low level to moderate of certainty.

### Implications of findings

Some physical examination tests showed promising diagnostic accuracy. Sela et al.’s study showed that the MC pressure-shear test is with the highest accuracy at 98% [22]. Gelberman et al.’s study showed that extension, adduction, adduction and extension, and adduction or extension also with very high accuracy, all exceeding 90% [15]. While Sela et al.’s study showed unclear risk of bias of index test and flow and timing, Gelberman et al.’s study showed low risk of bias on all domains. Comprehensively, the applicable concerns of these two studies are low, so these diagnostic tests could be recommended for use in clinical practice. Additionally, it’s important to note that the Grind test (53%∼76%) [13,15,20,22] and the Seesaw test (71%∼78%) [13], which are commonly used for diagnosing TB OA, did not demonstrate the highest diagnostic accuracy. This highlights the need for careful consideration when employing these diagnostic tests in clinical practice. We recommend that in clinical practice, not only should these two most used diagnostic tests be applied, but also consideration should be given to using the various diagnostic tests shown above to have higher diagnostic accuracy.

### Methods and limitations

This systematic review followed the rigorous methodology outlined in the PRISMA guideline and was registered on PROSPERO, ensuring transparency and credibility. The development of a comprehensive search strategy, in consultation with an expert librarian, included multiple databases to identify all relevant literature related to thumb OA diagnosis.

One importance choice was the focus on participants with hand or thumb pain, suspected of having thumb OA. This inclusion criterion ensured that the review closely aligned with the clinical reality faced by healthcare professionals who need reliable diagnostic tools for individuals exhibiting symptoms indicative of thumb OA.

Another noteworthy approach in this study was that female sex was considered as an index test, given the variation in the prevalence of thumb OA between males and females [32]. It should be noted that using female sex as an index test was not the original intent of the two articles that focus on the study of female sex. Although the data from these two articles do not show satisfactory diagnostic accuracy for female sex, it is worthwhile to conduct research on the diagnostic accuracy of using ‘female sex’ as an index test in the future. While the included studies have reported the overall age of the study population (as shown in Table 1), we could not calculate the diagnostic accuracy because the age distribution of patients and non-patients was unavailable. It is worthwhile to explore the diagnostic accuracy of utilizing ‘age distribution’ as an index test in future research or to investigate the accuracy of combining age with other tests as a diagnostic test.

Compared to radiographs, MR images may exhibit greater sensitivity in detecting TB OA [23]. In the only eligible study evaluating MRI [16], its original design aimed to analyze the MRI signal changes in individuals with thumb OA. In this study, the diagnostic criteria for thumb CMC OA were defined as Eaton stage ≥ 1. However, we included this study in the review because in some of other studies, Eaton stage = 1 was considered negative, and Eaton stage ≥ 2 was considered positive for CMC OA [20,22]. Therefore, to maintain comparability with other diagnostic modalities, X-ray with Eaton stage ≥2 was adopted as the reference standard while MRI signal changes as the index test. We conducted calculations according to the index test and reference standard adopted, and MRI signal changes exhibited a moderate level of diagnostic accuracy at 76%.

Not distinguishing the setting of the studies, whether they were conducted in primary care or secondary care settings is one limitation of this study. Neglecting to account for these variations can compromise the review’s generalizability to specific clinical contexts.

### Diagnostic accuracy and risk factors

A significant risk factor does not make an accurate diagnostic tool, per se. Occupations involving repetitive thumb use and insufficient rest breaks have been identified as significant risk factors for thumb CMC OA in women [33]. Additionally, manual labor of bank employees was a risk factor for TB OA [34]. Furthermore, in females, typing and dexterity-related work increased the risk of thumb CMC OA [35]. Moreover, men engaged in heavy manual work have been shown to have significantly higher odds ratios for thumb CMC OA compared to those involved in light work [36]. Similarly, women showed an elevated risk of CMC OA associated with occupational manual load, particularly in the light-moderate (OR: 1.46), moderate (OR: 1.27), and heavy (OR: 1.31) work categories [36]. However, the overall accuracy of manual occupation as a method for diagnosis in the current systematic review was found to be relatively low (46%∼50%) [17]. Apart from occupations and female sex, other studies studied several demographic variables as diagnostic factors, such as age, body mass index, and family history [37]. The classification criteria for knee OA and hip OA both mention the factor of age [38,39], while in the criteria for hand joint OA, age is not mentioned as a factor in the criteria for hand OA [40]. In a research on the criteria for hand OA, age showed an acceptable discriminatory capacity [37,41].

### Limited evidence for erosive thumb OA

Erosive hand OA is characterized by centrally located subchondral erosions, which have the potential to progress into significant bone and cartilage loss, resulting in joint instability and eventual bony ankylosis [9]. Erosive thumb OA was characterized by the existence of erosive or remodelled phases (Verbruggen–Veys classification [42]) in at least one interphalangeal joint or carpometacarpal joint [17]. There is limited evidence available specifically regarding erosive thumb OA, with only one study [17] included in this regard. Although erosive hand OA is more commonly seen in DIP joints, it is important to note that approximately 30% of individuals with erosive hand OA also exhibit erosions in the first CMCs [43]. Furthermore, the study provided evidence that erosive hand OA is more prevalent in females and is associated with a higher degree of pain compared to non-erosive hand OA [43]. However, our review indicated that relying solely on history taking items such as female sex, presence of TB pain, and manual occupation may have a relatively low accuracy, ranging from 40% to 50%, in identifying erosive CMC OA. This indicates the need for additional diagnostic approaches beyond these factors to accurately diagnose erosive CMC OA. Notably, ultrasound has been reported as a reliable and more sensitive imaging technique compared to conventional radiography in detecting erosions and osteophytes in individuals diagnosed with erosive hand OA [44], but in current review no study was identified.

### Future research directions

Future research should first focus on achieving consensus on diagnostic criteria, aiming to establish clear and widely accepted standards for diagnosing thumb OA that go beyond merely relying on structural changes. This includes considering clinical diagnosis and other relevant factors to improve the accuracy and effectiveness of the diagnostic process. By establishing universally accepted diagnostic guidelines, researchers can improve the consistency and comparability of diagnostic accuracy research in this field. Additionally, there is ample room for development in the diagnosis of individual thumb joints, necessitating further research and exploration. Exploring advanced imaging techniques such as computed tomography holds great promise in enhancing diagnostic capabilities for thumb OA [45]. These research directions will contribute to improved accuracy and standardization in the diagnosis of this condition.

While the findings of this systematic review highlight the differences in diagnostic accuracy among various methods, it is crucial to acknowledge that in clinical practice, the collection of medical history, physical examination, and auxiliary investigations should be viewed as interdependent and reinforcing components in clinical decision-making. Integrating these diagnostic approaches can lead to a more comprehensive assessment and enhance diagnostic accuracy for thumb OA. In future research, it is imperative to further integrate these diagnostic methods to achieve a more comprehensive evaluation and improve diagnostic accuracy.

## Conclusion

In conclusion, there is a limited amount of evidence regarding the diagnostic accuracy of history taking, physical examination, and auxiliary examination for diagnosing thumb OA. Furthermore, there is a lack of unified diagnostic criteria for thumb OA. Based on the limited evidence the adduction test, extension test, and MC pressure-shear test are currently the most accurate methods for diagnosing radiographic thumb OA, and the Grind and Seesaw test, did not demonstrate a high diagnostic accuracy.

## Supplementary Material

Supplemental Material

## Data Availability

All data generated or analyzed during this study are included in this article [and its Online Appendices].

## Other information

Registration and protocol: this systematic review has been registered on PROSPERO (Registration number: CRD42022309959)
