# Supplementary material for: Diagnostic accuracy of history taking, physical examination, and auxiliary examination for thumb osteoarthritis: a systematic review
Source: Ann Med. 2025 Jun 26;57(1):2524086. doi: 10.1080/07853890.2025.2524086 (PMC12551402; doi:10.1080/07853890.2025.2524086)
Supplement: Supplemental Material [file IANN_A_2524086_SM1341.zip › suppl_data/Appendix C Search strategy.docx]

Appendix C Search strategy

A total of three searches were conducted: the first on January 13, 2022, the second on March 11, 2022, and the third on October 5, 2023. The third search was the final one.

20220113

| **Database searched** | **via** | **Years of coverage** | **Records** | **Records after duplicates removed** |
| --- | --- | --- | --- | --- |
| Embase | Embase.com | 1971 - Present | 877 | 455 |
| Medline ALL | Ovid | 1946 - Present | 1217 | 1215 |
| Web of Science Core Collection* | Web of Knowledge | 1975 - Present | 91 | 9 |
| Cochrane Central Register of Controlled Trials | Wiley | 1992 - Present | 206 | 50 |
| CINAHL |  |  | 302 | 126 |
| **Total** | | | **2693** | **1855** |

*Science Citation Index Expanded (1975-present) ; Social Sciences Citation Index (1975-present) ; Arts & Humanities Citation Index (1975-present) ; Conference Proceedings Citation Index- Science (1990-present) ; Conference Proceedings Citation Index- Social Science & Humanities (1990-present) ; Emerging Sources Citation Index (2015-present)

**Embase.com 875**

('thumb osteoarthritis'/de OR 'trapeziometacarpal osteoarthritis'/de OR 'trapeziometacarpal arthritis'/de OR 'trapeziometacarpal joint osteoarthritis'/de OR rhizarthrosis/de OR (('osteoarthritis'/de OR 'hand osteoarthritis'/de OR arthritis/de OR 'rheumatoid arthritis'/de OR 'trapeziometacarpal arthritis'/de) AND (thumb/de OR 'thumb injury'/de OR 'trapeziometacarpal joint'/de)) OR ((thumb* OR first-carp* OR trapez* OR first-CMC* OR first-metacarp*) NEAR/6 (osteoarthrit* OR arthritis OR arthrosis OR arthroses) OR Rhizarthros*):Ab,ti OR ((thumb* OR first-carp* OR trapez* OR first-CMC* OR first-metacarp*) AND (osteoarthrit* OR arthritis OR arthrosis OR arthroses)):ti) AND (anamnesis/de OR 'physical examination'/de OR 'diagnostic accuracy'/exp OR 'diagnostic test'/de OR 'diagnostic value'/de OR 'diagnostic error'/exp OR 'sensitivity and specificity'/de OR 'diagnostic test accuracy study'/de OR 'musculoskeletal diagnosis'/de OR 'interrater reliability'/de OR radiodiagnosis/exp OR ultrasound/de OR 'kapandji score'/de OR goniometry/de OR goniometer/de OR diagnosis/exp/mj OR 'osteoarthritis'/exp/dm_di/mj OR 'flexion test'/de OR (((diagnos* OR detect* OR test*) NEAR/6 (accura* OR value* OR test OR error* OR missed OR evaluat*)) OR misdiagnos* OR undetect* OR (false NEXT/1 (positive* OR negative*)) OR sensitivity OR specificity OR validity OR reliabilit* OR precision OR interrater* OR intertester* OR interevaluat* OR interobserver* OR inter-rater* OR inter-tester* OR inter-evaluat* OR inter-observer* OR intrarater* OR intratester* OR intraevaluat* OR intraobserver* OR intra-rater* OR intra-tester* OR intra-evaluat* OR intra-observer* OR anamnesis* OR (history NEAR/3 taking) OR ((physical* OR auxiliar* OR clinical*) NEAR/3 examin*) OR ((extension* OR provocati* OR flexion OR adduction* OR compression* OR grind* OR pressure* OR shear* OR traction OR shift OR subluxation OR relocation OR strength OR dexterity) NEAR/6 test*) OR radiodiagnos* OR radiogra* OR (diagnos* NEAR/3 imag*) OR ultrasound* OR ultrasonog* OR mri OR (magnet* NEAR/3 resonan*) OR tomogra* OR Kapandji OR goniomet* OR Seesaw):ab,ti OR (diagnos* OR us OR ct OR grind* OR pressure* OR shear*):ti) NOT (surgery/exp/mj OR (surger* OR surgical*):ti) NOT ([animals]/lim NOT [humans]/lim)

**Medline ALL**

(((Osteoarthritis/ OR Arthritis/ OR Arthritis, Rheumatoid/) AND (Thumb/)) OR ((thumb* OR first-carp* OR trapez* OR first-CMC* OR first-metacarp*) ADJ6 (osteoarthrit* OR arthritis OR arthrosis OR arthroses) OR Rhizarthros*).ab,ti. OR ((thumb* OR first-carp* OR trapez* OR first-CMC* OR first-metacarp*) AND (osteoarthrit* OR arthritis OR arthrosis OR arthroses)).ti.) AND (Physical Examination/ OR exp Diagnostic Tests, Routine/ OR exp Diagnostic Errors/ OR "Sensitivity and Specificity"/ OR exp Ultrasonography/ OR Arthrometry, Articular/ OR * Diagnosis/ OR exp * Osteoarthritis/ OR (((diagnos* OR detect* OR test*) ADJ6 (accura* OR value* OR test OR error* OR missed OR evaluat*)) OR misdiagnos* OR undetect* OR (false ADJ (positive* OR negative*)) OR sensitivity OR specificity OR validity OR reliabilit* OR precision OR interrater* OR intertester* OR interevaluat* OR interobserver* OR inter-rater* OR inter-tester* OR inter-evaluat* OR inter-observer* OR intrarater* OR intratester* OR intraevaluat* OR intraobserver* OR intra-rater* OR intra-tester* OR intra-evaluat* OR intra-observer* OR anamnesis* OR (history ADJ3 taking) OR ((physical* OR auxiliar* OR clinical*) ADJ3 examin*) OR ((extension* OR provocati* OR flexion OR adduction* OR compression* OR grind* OR pressure* OR shear* OR traction OR shift OR subluxation OR relocation OR strength OR dexterity) ADJ6 test*) OR radiodiagnos* OR radiogra* OR (diagnos* ADJ3 imag*) OR ultrasound* OR ultrasonog* OR mri OR (magnet* ADJ3 resonan*) OR tomogra* OR Kapandji OR goniomet* OR Seesaw).ab,ti. OR (diagnos* OR us OR ct OR grind* OR pressure* OR shear*).ti.) NOT (* General Surgery/ OR (surger* OR surgical*).ti.) NOT (exp animals/ NOT humans/)

**Web of Science Core Collection***

TI=((((thumb* OR first-carp* OR trapez* OR first-CMC* OR first-metacarp*) NEAR/5 (osteoarthrit* OR arthritis OR arthrosis OR arthroses) OR Rhizarthros*) OR ((thumb* OR first-carp* OR trapez* OR first-CMC* OR first-metacarp*))) AND (osteoarthrit* OR arthritis OR arthrosis OR arthroses) AND ((((diagnos* OR detect* OR test*) NEAR/5 (accura* OR value* OR test OR error* OR missed OR evaluat*)) OR misdiagnos* OR undetect* OR (false NEAR/1 (positive* OR negative*)) OR sensitivity OR specificity OR validity OR reliabilit* OR precision OR interrater* OR intertester* OR interevaluat* OR interobserver* OR inter-rater* OR inter-tester* OR inter-evaluat* OR inter-observer* OR intrarater* OR intratester* OR intraevaluat* OR intraobserver* OR intra-rater* OR intra-tester* OR intra-evaluat* OR intra-observer* OR anamnesis* OR (history NEAR/2 taking) OR ((physical* OR auxiliar* OR clinical*) NEAR/2 examin*) OR ((extension* OR provocati* OR flexion OR adduction* OR compression* OR grind* OR pressure* OR shear* OR traction OR shift OR subluxation OR relocation OR strength OR dexterity) NEAR/5 test*) OR radiodiagnos* OR radiogra* OR (diagnos* NEAR/2 imag*) OR ultrasound* OR ultrasonog* OR mri OR (magnet* NEAR/2 resonan*) OR tomogra* OR Kapandji OR goniomet* OR Seesaw) OR (diagnos* OR us OR ct OR grind* OR pressure* OR shear*)) NOT (surger* OR surgical*) NOT (animals NOT humans))

**Cochrane Central Register of Controlled Trials**

(((thumb* OR first NEXT carp* OR trapez* OR first NEXT CMC* OR first NEXT metacarp*) NEAR/6 (osteoarthrit* OR arthritis OR arthrosis OR arthroses) OR Rhizarthros*):Ab,ti OR ((thumb* OR first NEXT carp* OR trapez* OR first NEXT CMC* OR first NEXT metacarp*) AND (osteoarthrit* OR arthritis OR arthrosis OR arthroses)):ti) AND ((((diagnos* OR detect* OR test*) NEAR/6 (accura* OR value* OR test OR error* OR missed OR evaluat*)) OR misdiagnos* OR undetect* OR (false NEXT/1 (positive* OR negative*)) OR sensitivity OR specificity OR validity OR reliabilit* OR precision OR interrater* OR intertester* OR interevaluat* OR interobserver* OR inter NEXT rater* OR inter NEXT tester* OR inter NEXT evaluat* OR inter NEXT observer* OR intrarater* OR intratester* OR intraevaluat* OR intraobserver* OR intra NEXT rater* OR intra NEXT tester* OR intra NEXT evaluat* OR intra NEXT observer* OR anamnesis* OR (history NEAR/3 taking) OR ((physical* OR auxiliar* OR clinical*) NEAR/3 examin*) OR ((extension* OR provocati* OR flexion OR adduction* OR compression* OR grind* OR pressure* OR shear* OR traction OR shift OR subluxation OR relocation OR strength OR dexterity) NEAR/6 test*) OR radiodiagnos* OR radiogra* OR (diagnos* NEAR/3 imag*) OR ultrasound* OR ultrasonog* OR mri OR (magnet* NEAR/3 resonan*) OR tomogra* OR Kapandji OR goniomet* OR Seesaw):ab,ti OR (diagnos* OR us OR ct OR grind* OR pressure* OR shear*):ti) NOT ((surger* OR surgical*):ti) NOT (animals NOT humans)

**CINAHL**

(((MH Osteoarthritis OR MH Arthritis OR MH Arthritis, Rheumatoid) AND (MH Thumb)) OR TI ((thumb* OR first-carp* OR trapez* OR first-CMC* OR first-metacarp*) N5 (osteoarthrit* OR arthritis OR arthrosis OR arthroses) OR Rhizarthros*) OR ((thumb* OR first-carp* OR trapez* OR first-CMC* OR first-metacarp*) AND (osteoarthrit* OR arthritis OR arthrosis OR arthroses))) AND (MH Physical Examination OR MH Diagnostic Tests, Routine OR MH Diagnostic Errors OR MH "Sensitivity and Specificity" OR MH Ultrasonography OR MH Arthrometry, Articular OR MM Diagnosis OR MM Osteoarthritis OR TI (((diagnos* OR detect* OR test*) N5 (accura* OR value* OR test OR error* OR missed OR evaluat*)) OR misdiagnos* OR undetect* OR (false N1 (positive* OR negative*)) OR sensitivity OR specificity OR validity OR reliabilit* OR precision OR interrater* OR intertester* OR interevaluat* OR interobserver* OR inter-rater* OR inter-tester* OR inter-evaluat* OR inter-observer* OR intrarater* OR intratester* OR intraevaluat* OR intraobserver* OR intra-rater* OR intra-tester* OR intra-evaluat* OR intra-observer* OR anamnesis* OR (history N2 taking) OR ((physical* OR auxiliar* OR clinical*) N2 examin*) OR ((extension* OR provocati* OR flexion OR adduction* OR compression* OR grind* OR pressure* OR shear* OR traction OR shift OR subluxation OR relocation OR strength OR dexterity) N5 test*) OR radiodiagnos* OR radiogra* OR (diagnos* N2 imag*) OR ultrasound* OR ultrasonog* OR mri OR (magnet* N2 resonan*) OR tomogra* OR Kapandji OR goniomet* OR Seesaw) OR (diagnos* OR us OR ct OR grind* OR pressure* OR shear*)) NOT (MM General Surgery OR (surger* OR surgical*)) NOT (MH animals NOT humans)

20220311

| **Database searched** | **via** | **Years of coverage** | **Records** | **Records after duplicates removed** |
| --- | --- | --- | --- | --- |
| Embase | Embase.com | 1971 - Present | 1007 | 995 |
| Medline ALL | Ovid | 1946 - Present | 703 | 329 |
| Web of Science Core Collection* | Web of Knowledge | 1975 - Present | 593 | 124 |
| Cochrane Central Register of Controlled Trials | Wiley | 1992 - Present | 209 | 27 |
| CINAHL | EBSCOhost |  | 307 | 158 |
| **Total** | | | **2819** | **1633** |

*Science Citation Index Expanded (1975-present) ; Social Sciences Citation Index (1975-present) ; Arts & Humanities Citation Index (1975-present) ; Conference Proceedings Citation Index- Science (1990-present) ; Conference Proceedings Citation Index- Social Science & Humanities (1990-present) ; Emerging Sources Citation Index (2015-present)

**Embase.com**

('thumb osteoarthritis'/de OR 'trapeziometacarpal osteoarthritis'/de OR 'trapeziometacarpal arthritis'/de OR 'trapeziometacarpal joint osteoarthritis'/de OR rhizarthrosis/de OR 'scaphotrapeziotrapezoid arthritis'/de OR 'scaphotrapeziotrapezoid osteoarthritis'/de OR (('osteoarthritis'/de OR 'hand osteoarthritis'/de OR arthritis/de OR 'rheumatoid arthritis'/de OR 'trapeziometacarpal arthritis'/de) AND (thumb/de OR 'thumb injury'/de OR 'trapeziometacarpal joint'/de OR 'scaphotrapeziotrapezoid joint'/de OR 'sesamoid bone'/de)) OR ((thumb* OR first-carp* OR trapez* OR scaphotrapez* OR first-CMC* OR stt OR triscaphe OR first-metacarp* OR sesamoid*) NEAR/6 (osteoarthrit* OR arthritis OR arthrosis OR arthroses) OR Rhizarthros*):Ab,ti OR ((thumb* OR first-carp* OR trapez* OR first-CMC* OR first-metacarp*) AND (osteoarthrit* OR arthritis OR arthrosis OR arthroses)):ti) AND (anamnesis/de OR 'physical examination'/de OR 'diagnostic accuracy'/exp OR 'diagnostic test'/de OR 'diagnostic value'/de OR 'diagnostic error'/exp OR 'sensitivity and specificity'/de OR 'diagnostic test accuracy study'/de OR 'musculoskeletal diagnosis'/de OR 'interrater reliability'/de OR radiodiagnosis/exp OR ultrasound/de OR 'kapandji score'/de OR goniometry/de OR goniometer/de OR diagnosis/exp/mj OR 'osteoarthritis'/exp/dm_di/mj OR 'flexion test'/de OR (((diagnos* OR detect* OR test*) NEAR/6 (accura* OR value* OR test OR error* OR missed OR evaluat*)) OR misdiagnos* OR undetect* OR (false NEXT/1 (positive* OR negative*)) OR sensitivity OR specificity OR validity OR reliabilit* OR precision OR interrater* OR intertester* OR interevaluat* OR interobserver* OR inter-rater* OR inter-tester* OR inter-evaluat* OR inter-observer* OR intrarater* OR intratester* OR intraevaluat* OR intraobserver* OR intra-rater* OR intra-tester* OR intra-evaluat* OR intra-observer* OR anamnesis* OR (history NEAR/3 taking) OR ((physical* OR auxiliar* OR clinical*) NEAR/3 examin*) OR ((extension* OR provocati* OR flexion OR adduction* OR compression* OR grind* OR pressure* OR shear* OR traction OR shift OR subluxation OR relocation OR strength OR dexterity) NEAR/6 test*) OR radiodiagnos* OR radiogra* OR (diagnos* NEAR/3 imag*) OR ultrasound* OR ultrasonog* OR mri OR (magnet* NEAR/3 resonan*) OR tomogra* OR Kapandji OR goniomet* OR Seesaw):ab,ti OR (diagnos* OR us OR ct OR grind* OR pressure* OR shear*):ti) NOT (surgery/exp/mj OR (surger* OR surgical*):ti) NOT ([animals]/lim NOT [humans]/lim)

**Medline ALL**

(((Osteoarthritis/ OR Arthritis/ OR Arthritis, Rheumatoid/ OR Sesamoid Bones/) AND (Thumb/)) OR (((thumb* OR first-carp* OR trapez* OR scaphotrapez* OR first-CMC* OR stt OR triscaphe OR first-metacarp* OR sesamoid*) ADJ6 (osteoarthrit* OR arthritis OR arthrosis OR arthroses)) OR Rhizarthros*).ab,ti. OR ((thumb* OR first-carp* OR trapez* OR first-CMC* OR first-metacarp*) AND (osteoarthrit* OR arthritis OR arthrosis OR arthroses)).ti.) AND (Physical Examination/ OR exp Diagnostic Tests, Routine/ OR exp Diagnostic Errors/ OR "Sensitivity and Specificity"/ OR exp Ultrasonography/ OR Arthrometry, Articular/ OR * Diagnosis/ OR exp * Osteoarthritis/di OR (((diagnos* OR detect* OR test*) ADJ6 (accura* OR value* OR test OR error* OR missed OR evaluat*)) OR misdiagnos* OR undetect* OR (false ADJ (positive* OR negative*)) OR sensitivity OR specificity OR validity OR reliabilit* OR precision OR interrater* OR intertester* OR interevaluat* OR interobserver* OR inter-rater* OR inter-tester* OR inter-evaluat* OR inter-observer* OR intrarater* OR intratester* OR intraevaluat* OR intraobserver* OR intra-rater* OR intra-tester* OR intra-evaluat* OR intra-observer* OR anamnesis* OR (history ADJ3 taking) OR ((physical* OR auxiliar* OR clinical*) ADJ3 examin*) OR ((extension* OR provocati* OR flexion OR adduction* OR compression* OR grind* OR pressure* OR shear* OR traction OR shift OR subluxation OR relocation OR strength OR dexterity) ADJ6 test*) OR radiodiagnos* OR radiogra* OR (diagnos* ADJ3 imag*) OR ultrasound* OR ultrasonog* OR mri OR (magnet* ADJ3 resonan*) OR tomogra* OR Kapandji OR goniomet* OR Seesaw).ab,ti. OR (diagnos* OR us OR ct OR grind* OR pressure* OR shear*).ti.) NOT (* General Surgery/ OR (surger* OR surgical*).ti.) NOT (exp animals/ NOT humans/)

**Web of Science Core Collection***

TS=((((thumb* OR first-carp* OR trapez* OR scaphotrapez* OR first-CMC* OR stt OR triscaphe OR first-metacarp* OR sesamoid*) NEAR/5 (osteoarthrit* OR arthritis OR arthrosis OR arthroses) OR Rhizarthros*) OR ((thumb* OR first-carp* OR trapez* OR first-CMC* OR first-metacarp*) AND (osteoarthrit* OR arthritis OR arthrosis OR arthroses)):ti) AND ((((diagnos* OR detect* OR test*) NEAR/5 (accura* OR value* OR test OR error* OR missed OR evaluat*)) OR misdiagnos* OR undetect* OR (false NEAR/1 (positive* OR negative*)) OR sensitivity OR specificity OR validity OR reliabilit* OR precision OR interrater* OR intertester* OR interevaluat* OR interobserver* OR inter-rater* OR inter-tester* OR inter-evaluat* OR inter-observer* OR intrarater* OR intratester* OR intraevaluat* OR intraobserver* OR intra-rater* OR intra-tester* OR intra-evaluat* OR intra-observer* OR anamnesis* OR (history NEAR/2 taking) OR ((physical* OR auxiliar* OR clinical*) NEAR/2 examin*) OR ((extension* OR provocati* OR flexion OR adduction* OR compression* OR grind* OR pressure* OR shear* OR traction OR shift OR subluxation OR relocation OR strength OR dexterity) NEAR/5 test*) OR radiodiagnos* OR radiogra* OR (diagnos* NEAR/2 imag*) OR ultrasound* OR ultrasonog* OR mri OR (magnet* NEAR/2 resonan*) OR tomogra* OR Kapandji OR goniomet* OR Seesaw))) NOT TI=(surger* OR surgical*)

**Cochrane Central Register of Controlled Trials**

(((thumb* OR first-carp* OR trapez* OR scaphotrapez* OR first-CMC* OR stt OR triscaphe OR first-metacarp* OR sesamoid*) NEAR/6 (osteoarthrit* OR arthritis OR arthrosis OR arthroses) OR Rhizarthros*):Ab,ti OR ((thumb* OR first-carp* OR trapez* OR first-CMC* OR first-metacarp*) AND (osteoarthrit* OR arthritis OR arthrosis OR arthroses)):ti) AND ((((diagnos* OR detect* OR test*) NEAR/6 (accura* OR value* OR test OR error* OR missed OR evaluat*)) OR misdiagnos* OR undetect* OR (false NEXT/1 (positive* OR negative*)) OR sensitivity OR specificity OR validity OR reliabilit* OR precision OR interrater* OR intertester* OR interevaluat* OR interobserver* OR inter-rater* OR inter-tester* OR inter-evaluat* OR inter-observer* OR intrarater* OR intratester* OR intraevaluat* OR intraobserver* OR intra-rater* OR intra-tester* OR intra-evaluat* OR intra-observer* OR anamnesis* OR (history NEAR/3 taking) OR ((physical* OR auxiliar* OR clinical*) NEAR/3 examin*) OR ((extension* OR provocati* OR flexion OR adduction* OR compression* OR grind* OR pressure* OR shear* OR traction OR shift OR subluxation OR relocation OR strength OR dexterity) NEAR/6 test*) OR radiodiagnos* OR radiogra* OR (diagnos* NEAR/3 imag*) OR ultrasound* OR ultrasonog* OR mri OR (magnet* NEAR/3 resonan*) OR tomogra* OR Kapandji OR goniomet* OR Seesaw):ab,ti OR (diagnos* OR us OR ct OR grind* OR pressure* OR shear*):ti) NOT ((surger* OR surgical*):ti)

**CINAHL**

(((MH Osteoarthritis OR MH Arthritis OR MH Arthritis, Rheumatoid) AND (MH Thumb)) OR TI ((thumb* OR first-carp* OR trapez* OR first-CMC* OR first-metacarp*) N5 (osteoarthrit* OR arthritis OR arthrosis OR arthroses) OR Rhizarthros*) OR ((thumb* OR first-carp* OR trapez* OR first-CMC* OR first-metacarp*) AND (osteoarthrit* OR arthritis OR arthrosis OR arthroses))) AND (MH Physical Examination OR MH Diagnostic Tests, Routine OR MH Diagnostic Errors OR MH "Sensitivity and Specificity" OR MH Ultrasonography OR MH Arthrometry, Articular OR MM Diagnosis OR MM Osteoarthritis OR TI (((diagnos* OR detect* OR test*) N5 (accura* OR value* OR test OR error* OR missed OR evaluat*)) OR misdiagnos* OR undetect* OR (false N1 (positive* OR negative*)) OR sensitivity OR specificity OR validity OR reliabilit* OR precision OR interrater* OR intertester* OR interevaluat* OR interobserver* OR inter-rater* OR inter-tester* OR inter-evaluat* OR inter-observer* OR intrarater* OR intratester* OR intraevaluat* OR intraobserver* OR intra-rater* OR intra-tester* OR intra-evaluat* OR intra-observer* OR anamnesis* OR (history N2 taking) OR ((physical* OR auxiliar* OR clinical*) N2 examin*) OR ((extension* OR provocati* OR flexion OR adduction* OR compression* OR grind* OR pressure* OR shear* OR traction OR shift OR subluxation OR relocation OR strength OR dexterity) N5 test*) OR radiodiagnos* OR radiogra* OR (diagnos* N2 imag*) OR ultrasound* OR ultrasonog* OR mri OR (magnet* N2 resonan*) OR tomogra* OR Kapandji OR goniomet* OR Seesaw) OR (diagnos* OR us OR ct OR grind* OR pressure* OR shear*)) NOT (MM General Surgery OR (surger* OR surgical*)) NOT (MH animals NOT humans)

20231005

| **Database searched** | **Platform** | **Years of coverage** | **Records** | **Records after duplicates removed** |
| --- | --- | --- | --- | --- |
| Medline ALL | Ovid | 1946 - Present | 939 | 937 |
| Embase | Embase.com | 1971 - Present | 1444 | 881 |
| Web of Science Core Collection* | Web of Knowledge | 1975 - Present | 816 | 154 |
| Cochrane Central Register of Controlled Trials** | Wiley | 1992 - Present | 265 | 162 |
| CINAHL Plus | EBSCO | 1982 - Present | 509 | 297 |
| **Total** | | | **3973** | **2431** |

*Science Citation Index Expanded (1975-present) ; Social Sciences Citation Index (1975-present) ; Arts & Humanities Citation Index (1975-present) ; Conference Proceedings Citation Index- Science (1990-present) ; Conference Proceedings Citation Index- Social Science & Humanities (1990-present) ; Emerging Sources Citation Index (2005-present)

No other database limits were used than those specified in the search strategies

*new references: 834*

**Embase.com** 1444

('thumb osteoarthritis'/de OR 'trapeziometacarpal osteoarthritis'/de OR 'trapeziometacarpal arthritis'/de OR 'trapeziometacarpal joint osteoarthritis'/de OR rhizarthrosis/de OR 'scaphotrapeziotrapezoid arthritis'/de OR 'scaphotrapeziotrapezoid osteoarthritis'/de OR (('osteoarthritis'/de OR 'hand osteoarthritis'/de OR arthritis/de OR 'rheumatoid arthritis'/de OR 'trapeziometacarpal arthritis'/de) AND (thumb/de OR 'thumb injury'/de OR 'trapeziometacarpal joint'/de OR 'scaphotrapeziotrapezoid joint'/de OR 'sesamoid bone'/de OR 'carpometacarpal joint'/de OR 'interphalangeal joint of the hand'/de)) OR ((thumb* OR first-carp* OR trapez* OR scaphotrapez* OR first-CMC* OR stt OR triscaphe OR first-metacarp* OR sesamoid* OR CMC OR carpometacarp* OR STT ) NEAR/6 (osteoarthrit* OR arthritis OR arthrosis OR arthroses OR OA) OR Rhizarthros*):Ab,ti,kw OR ((thumb* OR first-carp* OR trapez* OR first-CMC* OR first-metacarp* OR CMC OR carpometacarp* OR (hand AND (interphalangeal-joint* OR IP)) OR STT) AND (osteoarthrit* OR arthritis OR arthrosis OR arthroses OR OA)):ti) **AND** (anamnesis/de OR 'physical examination'/de OR 'diagnostic accuracy'/exp OR 'diagnostic test'/de OR 'diagnostic value'/de OR 'diagnostic error'/exp OR 'sensitivity and specificity'/de OR 'diagnostic test accuracy study'/de OR 'musculoskeletal diagnosis'/de OR 'interrater reliability'/de OR radiodiagnosis/exp OR ultrasound/de OR 'kapandji score'/de OR goniometry/de OR goniometer/de OR diagnosis/exp/mj OR 'osteoarthritis'/exp/dm_di/mj OR 'flexion test'/de OR (((diagnos* OR detect* OR test*) NEAR/6 (accura* OR value* OR test OR error* OR missed OR evaluat*)) OR misdiagnos* OR undetect* OR (false NEXT/1 (positive* OR negative*)) OR sensitivity OR specificity OR validity OR reliabilit* OR precision OR interrater* OR intertester* OR interevaluat* OR interobserver* OR inter-rater* OR inter-tester* OR inter-evaluat* OR inter-observer* OR intrarater* OR intratester* OR intraevaluat* OR intraobserver* OR intra-rater* OR intra-tester* OR intra-evaluat* OR intra-observer* OR anamnesis* OR (history NEAR/3 taking) OR ((physical* OR auxiliar* OR clinical*) NEAR/3 examin*) OR ((extension* OR provocati* OR flexion OR adduction* OR compression* OR grind* OR pressure* OR shear* OR traction OR shift OR subluxation OR relocation OR strength OR dexterity) NEAR/6 test*) OR radiodiagnos* OR radiogra* OR (diagnos* NEAR/3 imag*) OR ultrasound* OR ultrasonog* OR mri OR (magnet* NEAR/3 resonan*) OR tomogra* OR Kapandji OR goniomet* OR Seesaw):ab,ti,kw OR (diagnos* OR us OR ct OR grind* OR pressure* OR shear*):ti) NOT (surgery/exp/mj OR (surger* OR surgical*):ti) NOT ([animals]/lim NOT [humans]/lim)

**Medline ALL**

(((Osteoarthritis/ OR Arthritis/ OR Arthritis, Rheumatoid/ OR Sesamoid Bones/) AND (Thumb/ OR Carpometacarpal Joints/)) OR (((thumb* OR first-carp* OR trapez* OR scaphotrapez* OR first-CMC* OR stt OR triscaphe OR first-metacarp* OR sesamoid* OR CMC OR carpometacarp* OR STT) ADJ6 (osteoarthrit* OR arthritis OR arthrosis OR arthroses OR OA)) OR Rhizarthros*).ab,ti,kf. OR ((thumb* OR first-carp* OR trapez* OR first-CMC* OR first-metacarp* OR CMC OR carpometacarp* OR (hand AND (interphalangeal-joint* OR IP)) OR STT) AND (osteoarthrit* OR arthritis OR arthrosis OR arthroses OR OA)).ti.) AND (Physical Examination/ OR exp Diagnostic Tests, Routine/ OR exp Diagnostic Errors/ OR "Sensitivity and Specificity"/ OR exp Ultrasonography/ OR Arthrometry, Articular/ OR * Diagnosis/ OR exp * Osteoarthritis/di OR (((diagnos* OR detect* OR test*) ADJ6 (accura* OR value* OR test OR error* OR missed OR evaluat*)) OR misdiagnos* OR undetect* OR (false ADJ (positive* OR negative*)) OR sensitivity OR specificity OR validity OR reliabilit* OR precision OR interrater* OR intertester* OR interevaluat* OR interobserver* OR inter-rater* OR inter-tester* OR inter-evaluat* OR inter-observer* OR intrarater* OR intratester* OR intraevaluat* OR intraobserver* OR intra-rater* OR intra-tester* OR intra-evaluat* OR intra-observer* OR anamnesis* OR (history ADJ3 taking) OR ((physical* OR auxiliar* OR clinical*) ADJ3 examin*) OR ((extension* OR provocati* OR flexion OR adduction* OR compression* OR grind* OR pressure* OR shear* OR traction OR shift OR subluxation OR relocation OR strength OR dexterity) ADJ6 test*) OR radiodiagnos* OR radiogra* OR (diagnos* ADJ3 imag*) OR ultrasound* OR ultrasonog* OR mri OR (magnet* ADJ3 resonan*) OR tomogra* OR Kapandji OR goniomet* OR Seesaw).ab,ti,kf. OR (diagnos* OR us OR ct OR grind* OR pressure* OR shear*).ti.) NOT (* General Surgery/ OR (surger* OR surgical*).ti.) NOT (exp animals/ NOT humans/)

**Web of Science Core Collection***

(TS=(((thumb* OR first-carp* OR trapez* OR scaphotrapez* OR first-CMC* OR stt OR triscaphe OR first-metacarp* OR sesamoid* OR CMC OR carpometacarp* OR STT) NEAR/5 (osteoarthrit* OR arthritis OR arthrosis OR arthroses OR OA)) OR Rhizarthros*) OR TI=((thumb* OR first-carp* OR trapez* OR first-CMC* OR first-metacarp* OR CMC OR carpometacarp* OR (hand AND (interphalangeal-joint* OR IP)) OR STT) AND (osteoarthrit* OR arthritis OR arthrosis OR arthroses OR OA))) AND TS=(((((diagnos* OR detect* OR test*) NEAR/5 (accura* OR value* OR test OR error* OR missed OR evaluat*)) OR misdiagnos* OR undetect* OR (false NEAR/1 (positive* OR negative*)) OR sensitivity OR specificity OR validity OR reliabilit* OR precision OR interrater* OR intertester* OR interevaluat* OR interobserver* OR inter-rater* OR inter-tester* OR inter-evaluat* OR inter-observer* OR intrarater* OR intratester* OR intraevaluat* OR intraobserver* OR intra-rater* OR intra-tester* OR intra-evaluat* OR intra-observer* OR anamnesis* OR (history NEAR/2 taking) OR ((physical* OR auxiliar* OR clinical*) NEAR/2 examin*) OR ((extension* OR provocati* OR flexion OR adduction* OR compression* OR grind* OR pressure* OR shear* OR traction OR shift OR subluxation OR relocation OR strength OR dexterity) NEAR/5 test*) OR radiodiagnos* OR radiogra* OR (diagnos* NEAR/2 imag*) OR ultrasound* OR ultrasonog* OR mri OR (magnet* NEAR/2 resonan*) OR tomogra* OR Kapandji OR goniomet* OR Seesaw))) NOT TI=(surger* OR surgical*)

**Cochrane Central Register of Controlled Trials**

(((thumb* OR first-carp* OR trapez* OR scaphotrapez* OR first-CMC* OR stt OR triscaphe OR first-metacarp* OR sesamoid* OR CMC OR carpometacarp* OR STT) NEAR/6 (osteoarthrit* OR arthritis OR arthrosis OR arthroses OR OA) OR Rhizarthros*):Ab,ti OR ((thumb* OR first-carp* OR trapez* OR first-CMC* OR first-metacarp*) AND (osteoarthrit* OR arthritis OR arthrosis OR arthroses OR OA)):ti) AND ((((diagnos* OR detect* OR test*) NEAR/6 (accura* OR value* OR test OR error* OR missed OR evaluat*)) OR misdiagnos* OR undetect* OR (false NEXT/1 (positive* OR negative*)) OR sensitivity OR specificity OR validity OR reliabilit* OR precision OR interrater* OR intertester* OR interevaluat* OR interobserver* OR inter-rater* OR inter-tester* OR inter-evaluat* OR inter-observer* OR intrarater* OR intratester* OR intraevaluat* OR intraobserver* OR intra-rater* OR intra-tester* OR intra-evaluat* OR intra-observer* OR anamnesis* OR (history NEAR/3 taking) OR ((physical* OR auxiliar* OR clinical*) NEAR/3 examin*) OR ((extension* OR provocati* OR flexion OR adduction* OR compression* OR grind* OR pressure* OR shear* OR traction OR shift OR subluxation OR relocation OR strength OR dexterity) NEAR/6 test*) OR radiodiagnos* OR radiogra* OR (diagnos* NEAR/3 imag*) OR ultrasound* OR ultrasonog* OR mri OR (magnet* NEAR/3 resonan*) OR tomogra* OR Kapandji OR goniomet* OR Seesaw):ab,ti OR (diagnos* OR us OR ct OR grind* OR pressure* OR shear*):ti) NOT ((surger* OR surgical*):ti)

**CINAHL**

(((MH Osteoarthritis OR MH Arthritis OR MH Arthritis, Rheumatoid) AND (MH Thumb)) OR TI ((thumb* OR first-carp* OR trapez* OR first-CMC* OR first-metacarp* OR CMC OR carpometacarp* OR (hand AND (interphalangeal-joint* OR IP)) OR STT) N5 (osteoarthrit* OR arthritis OR arthrosis OR arthroses OR OA) OR Rhizarthros*) OR ((thumb* OR first-carp* OR trapez* OR first-CMC* OR first-metacarp* OR CMC OR carpometacarp* OR (hand AND (interphalangeal-joint* OR IP)) OR STT) AND (osteoarthrit* OR arthritis OR arthrosis OR arthroses OR OA))) AND (MH Physical Examination OR MH Diagnostic Tests, Routine OR MH Diagnostic Errors OR MH "Sensitivity and Specificity" OR MH Ultrasonography OR MH Arthrometry, Articular OR MM Diagnosis OR MM Osteoarthritis OR TI (((diagnos* OR detect* OR test*) N5 (accura* OR value* OR test OR error* OR missed OR evaluat*)) OR misdiagnos* OR undetect* OR (false N1 (positive* OR negative*)) OR sensitivity OR specificity OR validity OR reliabilit* OR precision OR interrater* OR intertester* OR interevaluat* OR interobserver* OR inter-rater* OR inter-tester* OR inter-evaluat* OR inter-observer* OR intrarater* OR intratester* OR intraevaluat* OR intraobserver* OR intra-rater* OR intra-tester* OR intra-evaluat* OR intra-observer* OR anamnesis* OR (history N2 taking) OR ((physical* OR auxiliar* OR clinical*) N2 examin*) OR ((extension* OR provocati* OR flexion OR adduction* OR compression* OR grind* OR pressure* OR shear* OR traction OR shift OR subluxation OR relocation OR strength OR dexterity) N5 test*) OR radiodiagnos* OR radiogra* OR (diagnos* N2 imag*) OR ultrasound* OR ultrasonog* OR mri OR (magnet* N2 resonan*) OR tomogra* OR Kapandji OR goniomet* OR Seesaw) OR (diagnos* OR us OR ct OR grind* OR pressure* OR shear*)) NOT (MM General Surgery OR (surger* OR surgical*)) NOT (MH animals NOT humans)
