# Supplementary material for: Diagnostic accuracy of history taking, physical examination, and auxiliary examination for thumb osteoarthritis: a systematic review
Source: Ann Med. 2025 Jun 26;57(1):2524086. doi: 10.1080/07853890.2025.2524086 (PMC12551402; doi:10.1080/07853890.2025.2524086)
Supplement: Supplemental Material [file IANN_A_2524086_SM1341.zip › suppl_data/Appendix D Results of Methodological Assessment.docx]

Appendix D Results of Methodological Assessment

| Author, Year | Risk of Bias | | | | Applicability Concerns | | |
| --- | --- | --- | --- | --- | --- | --- | --- |
|  | Patient Selection | Index Test | Reference Standard | Flow and Timing | Patient Selection | Index Test | Reference Standard |
| Arnold et al., 2020 | ? | 😊 | 😟 | ? | 😊 | 😊 | 😊 |
| Gelberman et al., 2015 | 😊 | 😊 | 😊 | 😊 | 😊 | 😊 | 😊 |
| Merritt et al., 2010 | ? | 😊 | 😊 | 😊 | 😊 | 😊 | 😊 |
| Sela et al., 2019 | 😊 | ? | 😊 | ? | 😊 | 😊 | 😊 |
| Komatsu et al., 2017 | ? | ? | 😊 | ? | 😊 | ? | 😊 |
| Kwok et al., 2014 | 😊 | 😊 | 😊 | 😊 | 😊 | 😊 | 😊 |
| Model et al., 2016 | 😊 | 😊 | 😊 | 😊 | 😊 | 😊 | 😊 |

😊 =low risk; 😟 =high risk; ? =unclear risk.
