# Supplementary material for: Diagnostic accuracy of history taking, physical examination, and auxiliary examination for thumb osteoarthritis: a systematic review
Source: Ann Med. 2025 Jun 26;57(1):2524086. doi: 10.1080/07853890.2025.2524086 (PMC12551402; doi:10.1080/07853890.2025.2524086)
Supplement: Supplemental Material [file IANN_A_2524086_SM1341.zip › suppl_data/Appendix E Certainty of Evidence.docx]

Appendix E Certainty of Evidence

**Table-A Question**: Should history taking be used to diagnose thumb OA in symptomatic hand?

| \| Sensitivity \| 0.47 to 1.00 \| \| --- \| --- \| \| Specificity \| 0.40 to 0.63 \| |  | \| Prevalences \| 72% \| 2% \| 54% \| \| --- \| --- \| --- \| --- \| |  |
| --- | --- | --- | --- | --- | --- | --- | --- | --- | --- | --- | --- |

| Outcome | № of studies (№ of patients) | Study design | Factors that may decrease certainty of evidence | | | | | Effect per 1,000 patients tested | | | Test accuracy CoE |
| --- | --- | --- | --- | --- | --- | --- | --- | --- | --- | --- | --- |
|  |  |  | Risk of bias | Indirectness | Inconsistency | Imprecision | Publication bias | pre-test probability of72% | pre-test probability of2% | pre-test probability of54% |  |
| **True positives** (patients with thumb OA) | 2 studies 1096 patients | cohort & case-control type studies | serious | serious | serious | not serious | none | 338 to 720 | 9 to 20 | 254 to 540 | ⨁◯◯◯ Very low |
| **False negatives** (patients incorrectly classified as not having thumb OA) |  |  |  |  |  |  |  | 0 to 382 | 0 to 11 | 0 to 286 |  |
| **True negatives** (patients without thumb OA) | 2 studies 1096 patients | cohort & case-control type studies |  |  |  |  |  | 112 to 176 | 392 to 617 | 184 to 290 | - |
| **False positives** (patients incorrectly classified as having thumb OA) |  |  |  |  |  |  |  | 104 to 168 | 363 to 588 | 170 to 276 |  |

**Table-B Question**: Should physical examination be used to diagnose thumb OA in symptomatic hand?

| \| Sensitivity \| 0.02 to 1.00 \| \| --- \| --- \| \| Specificity \| 0.75 to 1.00 \| |  | \| Prevalences \| 35% \| 69% \| 83% \| \| --- \| --- \| --- \| --- \| |  |
| --- | --- | --- | --- | --- | --- | --- | --- | --- | --- | --- | --- |

| Outcome | № of studies (№ of patients) | Study design | Factors that may decrease certainty of evidence | | | | | Effect per 1,000 patients tested | | | Test accuracy CoE |
| --- | --- | --- | --- | --- | --- | --- | --- | --- | --- | --- | --- |
|  |  |  | Risk of bias | Indirectness | Inconsistency | Imprecision | Publication bias | pre-test probability of35% | pre-test probability of69% | pre-test probability of83% |  |
| **True positives** (patients with thumb OA) | 5 studies 427 patients | cohort & case-control type studies | not serious | not serious | serious^a^ | not serious | none | 7 to 350 | 14 to 690 | 17 to 830 | ⨁⨁⨁◯ Moderate |
| **False negatives** (patients incorrectly classified as not having thumb OA) |  |  |  |  |  |  |  | 0 to 343 | 0 to 676 | 0 to 813 |  |
| **True negatives** (patients without thumb OA) | 5 studies 427 patients | cohort & case-control type studies |  |  |  |  |  | 488 to 650 | 233 to 310 | 128 to 170 | - |
| **False positives** (patients incorrectly classified as having thumb OA) |  |  |  |  |  |  |  | 0 to 162 | 0 to 77 | 0 to 42 |  |

#### Explanations

a. A variety of physical examination methods and reference standards are used, and even studies that choose the same physical examination methods and reference standards report different results.

**Table-C Question**: Should auxiliary examination be used to diagnose thumb OA in symptomatic hand?

| \| Sensitivity \| 0.72 (95% CI: -- to --) \| \| --- \| --- \| \| Specificity \| 0.86 (95% CI: -- to --) \| |  | \| Prevalences \| 72% \|  \|  \| \| --- \| --- \| --- \| --- \| |  |
| --- | --- | --- | --- | --- | --- | --- | --- | --- | --- | --- | --- |

| Outcome | № of studies (№ of patients) | Study design | Factors that may decrease certainty of evidence | | | | | Effect per 1,000 patients tested | Test accuracy CoE |
| --- | --- | --- | --- | --- | --- | --- | --- | --- | --- |
|  |  |  | Risk of bias | Indirectness | Inconsistency | Imprecision | Publication bias | pre-test probability of72% |  |
| **True positives** (patients with thumb OA) | 1 studies 20 patients | cohort & case-control type studies | serious | serious | not serious | not serious | none | 518 (0 to 0) | ⨁⨁◯◯ Low |
| **False negatives** (patients incorrectly classified as not having thumb OA) |  |  |  |  |  |  |  | 202 (720 to 720) |  |
| **True negatives** (patients without thumb OA) | 1 studies 20 patients | cohort & case-control type studies |  |  |  |  |  | 241 (0 to 0) | - |
| **False positives** (patients incorrectly classified as having thumb OA) |  |  |  |  |  |  |  | 39 (280 to 280) |  |
